# Gonad-Sparing Surgical Sterilization in Dogs

**DOI:** 10.3389/fvets.2020.00342

**Published:** 2020-06-12

**Authors:** Michelle A. Kutzler

**Affiliations:** Department of Animal and Rangeland Sciences, Oregon State University, Corvallis, OR, United States

**Keywords:** mammary cancer, ovaries, ovary-sparing hysterectomy, testes, vasectomy

## Abstract

Elective sterilization of pet dogs is a common surgical procedure performed in veterinary practice. The main benefit of sterilization is population control and the reduction in euthanasia of unwanted dogs. The most common methods for sterilizing female and male dogs are ovariohysterectomy (spay; which removes both the ovaries and the uterus) and castration (neutering; which involves removing the testicles), respectively. However, any surgery that removes the gonads changes the animal in both positive and negative ways. There is mounting evidence supporting the long-term health complications associated with surgical sterilization with gonad removal. Gonads are not merely gamete-producing or ancillary sex/reproductive organs but rather they are necessary endocrine glands for normal metabolic, behavioral, musculoskeletal, and anti-neoplastic health. The purpose of this mini review is to describe two gonad sparing surgeries that this author has used to sterilize dogs. These surgeries can be performed on pediatric patients without interfering with pubertal maturation. Dog owners can make the decision when the dog is completely mature whether or not the gonads should be removed.

Ovary-sparing hysterectomy (OSH) is a gonad-sparing sterilization technique first described in the literature over 40 years ago (1). This surgery removes the entire uterus and all (or most) of the cervix but leaves the ovaries intact and functional (Figure 1). The surgical approach is like a spay but the incision longer and more caudal to facilitate greater access to remove the cervix. Starting with the left uterine horn, follow it cranially to the ovary. It is not necessary to breakdown the ovarian suspensory ligaments but doing so may improve visibility of the cranial aspects of the uterine horns. Make a window in the broad ligament adjacent to the ovary and uterine horn and then place a clamp across the mesosalpinx containing the uterine tube. A ligation is placed in the crush created by the clamp and the area between the ligature and the clamp is sharply transected. It is extremely important that all of the uterine horn adjacent to the ovary is removed and this area is carefully inspected before the ovary is returned to its abdominal location. The procedure is repeated for the right uterine horn. The broad ligaments are broken down toward the uterine body similar to a traditional ovariohysterectomy. Clamps are placed across the cranial vagina & circumferential or transfixing ligatures are placed in the crush.

**Figure 1:**
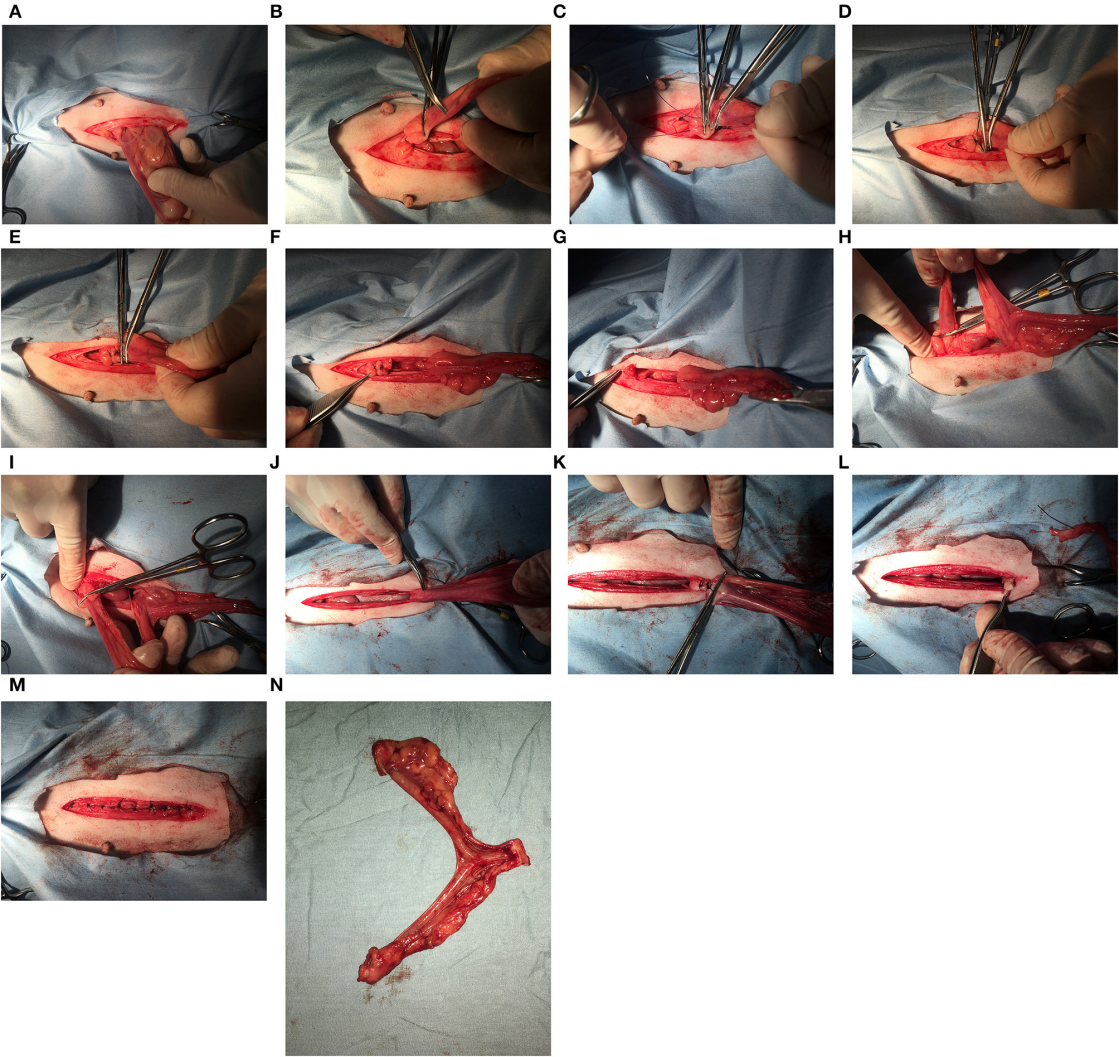
When the ovary-sparing hysterectomy is properly performed, the entire uterus and all or part of the cervix must be removed. For the procedure documented here, the cranial part of the cervix was removed. For the following series of images, the left of the image is cranial. **(A)** The left uterine horn is identified and elevated out of the incision. It is not necessary to breakdown the ovarian suspensory ligament but doing so may improve visibility of the cranial aspects of the uterine horns. **(B)** A hemostat has been placed caudal to the left ovary and the cranial aspect of the uterine horn is at the tip of the second hemostat. **(C)** A hemostat is placed cranial to the tip of the uterine horn, and two additional hemostats are placed between this hemostat and the ovary. **(D)** A transfixing suture is placed in the crush of the most cranial hemostat. **(E)** A second transfixing suture is placed in the crush of the next most cranial hemostat. **(F)** The left proper ligament and uterine tube are sharply transected caudal to the two transfixing sutures. **(G)** The left ovary is observed for signs of hemorrhage before replacing into the abdomen. **(H)** The right uterine horn is identified and elevated out of the incision. **(I)** A hemostat has been placed cranial to the right uterine horn and the process from the left side is repeated. **(J)** After breaking down the broad ligaments, the uterine horns, and body are elevated and reflected caudally to expose the cervix. It may be necessary to length the incision to elevate the entire cervix out of the incision. **(K)** Two transfixing sutures are placed around the vagina caudal to the cervix (or around the caudal aspect of cervix, where it meets the vagina as shown here). **(L)** The cranial vagina/caudal cervix is sharply transected and the entire uterus is removed. **(M)** Closure of the body wall, subcutaneous tissues and skin are same as with a routine ovariohysterectomy. **(N)** Removal of the entire uterus and cervix.

Some surgeons prefer to place clamps & ligate across the cervix but because of the cartilaginous nature of the canine cervix, it is the preference of this author to remove the entire cervix to ensure a more secure closure during ligation. The entire uterus must be removed, which is the reason for removing all (or at least part) of the cervix. It cannot be stressed enough that failure to completely remove the entire uterus will predispose for a pyometra. The closure of the abdominal and skin layers is similar to that of a routine ovariohysterectomy, which the exception that closure may take a little longer owing to a slightly longer incision.

Although the post-surgical recovery is also like a traditional ovariohysterectomy, bitches that have undergone an OSH will still experience routine estrous cycles and display an enlarged vulva (with no estrual bleeding because the uterus has been completely removed) and estrous behaviors (which includes a willingness to breed). Owners should be instructed to prevent females from mating until the vaginal closure is completely healed (~60 days). After this time, there is minimal risk of vaginal injury. However, there are a few anecdotal reports of life-threatening injury (e.g., cranial vaginal rupture) following mating in bitches following OSH and following traditional ovariohysterectomy with an ovarian remnant. Dogs undergoing OSH are presumably at a greater risk for developing mammary cancer than those who were routinely spayed. However, it is important to note that in at least one study, none of the 120 ovarian intact females developed mammary cancer but two of the spayed females did (2). This suggests that the link between mammary cancer and ovarian exposure in dogs should be re-examined (3). In addition, it should be noted that OSH performed in women has been associated with a premature reduction in circulating anti-Mullerian hormone (AMH) concentrations and to an earlier onset menopause (4). It is not known what, if any, effect performing an OSH in dogs will have on ovarian reserve so this is an area that warrants investigation.

Vasectomy involves bilateral removal and/or occlusion of a portion of the vas deferens, rendering the animal infertile by preventing sperm from being ejaculated during copulation. The quick procedure is technically like the surgery performed in men, with the exception that it is accomplished under general anesthesia in dogs. Following induction of general anesthesia, the inguinal region is prepared for surgery. A single small (1–3 cm) midline incision is made cranial to the scrotum. Some surgeons prefer to make one incision over each vas. Caudal traction placed on the scrotum will put tension on the spermatic cord, facilitating identification. The cord is dissected from the surrounding fat and fascia and a small incision is made through the parietal vaginal tunic. The testicular vessels lie in lateral fold of the visceral vaginal tunic, while the vas deferens with the deferential artery & vein lie in the medial fold. The vas deferens is isolated and the deferential artery and vein are dissected from the surface of the duct. Ligatures are placed on either side of a small section of vas deferens and the duct is removed between the ligatures. Recanalization has been reported when the duct is simply severed. The parietal vaginal tunic is closed with fine absorbable suture and the skin is closed with an absorbable suture in a subcuticular pattern. The procedure is then repeated on the opposite side.

According to Schiff et al. (5), sperm are completely absent from the ejaculate within 24 h following vasectomy. Male secondary sex characteristics and behaviors, as well as androgen-dependent diseases, are not prevented, since androgens are still produced. Following the vasectomy, increased intraluminal pressure within the rete testis may result in irreversible damage within the seminiferous cell population and testicular degeneration. In addition, the epididymis becomes distends with sperm. Epididymal fluid may extravasate under pressure, inducing a sperm granuloma. Up to 33% of vasectomized men experience chronic scrotal discomfort in the scrotum (6). Recurrent scrotal dermatitis (presumable associated with the discomfort) has been reported in dogs following vasectomy (7).

Both of these gonad sparing techniques are accepted as alternative surgical sterilization methods by the American Veterinary Medical Association (https://www.avma.org/public/petcare/pages/spay-neuter.aspx). Veterinarians and pet owners need to discuss which sterilization procedure is best for the long-term health of their pet.

## Author Contributions

The author confirms being the sole contributor of this work and has approved it for publication.

## Conflict of Interest

The author declares that the research was conducted in the absence of any commercial or financial relationships that could be construed as a potential conflict of interest.
